# Co-Occurrence of Aortic Stenosis and Coronary Artery Disease: Facing Challenges Before, During, and After Transcatheter Aortic Valve Replacement

**DOI:** 10.3390/jcm14134709

**Published:** 2025-07-03

**Authors:** Mihail Celeski, Annunziata Nusca, Nicolò Graziano Ciavaroli, Arianna Martucciello, Filippo Crisci, Dajana Polito, Fabio Mangiacapra, Valeria Cammalleri, Rosetta Melfi, Paolo Gallo, Elisabetta Ricottini, Nino Cocco, Raffaele Rinaldi, Annamaria Tavernese, Gian Paolo Ussia

**Affiliations:** 1 UOC Emodinamica, Fondazione Policlinico Universitario Campus Bio-Medico, Via Alvaro del Portillo, 200, 00128 Roma, Italyn.ciavaroli@unicampus.it (N.G.C.); v.cammalleri@policlinicocampus.it (V.C.);; 2 Unit of Experimental and Translational Cardiology, Department of Medicine and Surgery, Università Campus Bio-Medico di Roma, Via Alvaro del Portillo, 21, 00128 Roma, Italy

**Keywords:** aortic valve stenosis, transcatheter aortic valve replacement, transcatheter aortic valve implantation, coronary artery disease, percutaneous coronary intervention, commissural alignment, coronary access in TAVR

## Abstract

The introduction of transcatheter aortic valve replacement (TAVR) has revolutionized the management of aortic stenosis (AS), leading to significant improvements in patient outcomes. Over time, advancements in device technology have further optimized safety and performance of TAVR. However, as the pool of low-risk patients undergoing TAVR expands, many of whom present with concomitant coronary artery disease (CAD), new challenges have emerged. A large proportion of TAVR candidates suffer from CAD, and the clinical implications of this comorbidity remain a subject of debate. Research on the relationship between AS and CAD has yielded conflicting results, but severe CAD is generally linked to worse outcomes in AS patients. The coexistence of AS and CAD complicates diagnosis and management, requiring a comprehensive understanding of both invasive and non-invasive diagnostic techniques, along with careful revascularization strategies. This review explores the prevalence, clinical impact, and diagnostic challenges of CAD in TAVR patients, highlighting emerging methods for its assessment. Key aspects of treatment, including the timing of coronary revascularization, coronary re-access after TAVR in different settings, as well as practical tips and tricks for coronary cannulation, are also discussed. The complexity of managing AS and CAD is further intensified by the need for individualized approaches, particularly in hybrid procedures and subsequent TAVR interventions. Ongoing research and technological innovations offer promising solutions for refining the management of CAD in AS patients undergoing TAVR, with an emphasis on improving prognostic accuracy, optimizing revascularization strategies, and enhancing post-procedural care.

## 1. Introduction

In 2002, the management of aortic stenosis (AS) was completely revolutionized by the introduction of transcatheter aortic valve replacement (TAVR). Over the past 22 years, the development of TAVR has continued to evolve, moved by technological advancements that have driven continuous evolution in device design, including improvements in materials, structure, and prosthetic morphology, all aimed at ensuring optimal performance and safety for patients. However, implant after implant, the interventional cardiologist community has continually faced increasingly challenging obstacles. One of the most significant challenges in patients with severe AS is the concomitant presence of coronary artery disease (CAD). This issue is of upmost relevance, since the indications for treating low-risk patients are expanding, usually encompassing younger patients with longer life expectancies who may require coronary revascularization during follow-up. Given that AS and CAD share similar pathophysiological molecular pathways and risk factors, approximately one-half of TAVR candidates exhibit CAD. However, many observational studies have reported controversial results regarding the impact of CAD and its treatment in patients undergoing TAVR [1,2].

In this context, given the high likelihood of encountering patients with CAD undergoing TAVR, it also becomes essential to possess a thorough understanding of both invasive and non-invasive diagnostic options to assess myocardial ischemia and guide revascularization. Additionally, careful consideration must be given to revascularization strategies, including aspects such as the timing of revascularization, commissural alignment, and coronary re-access after TAVR.

**Figure 1:**
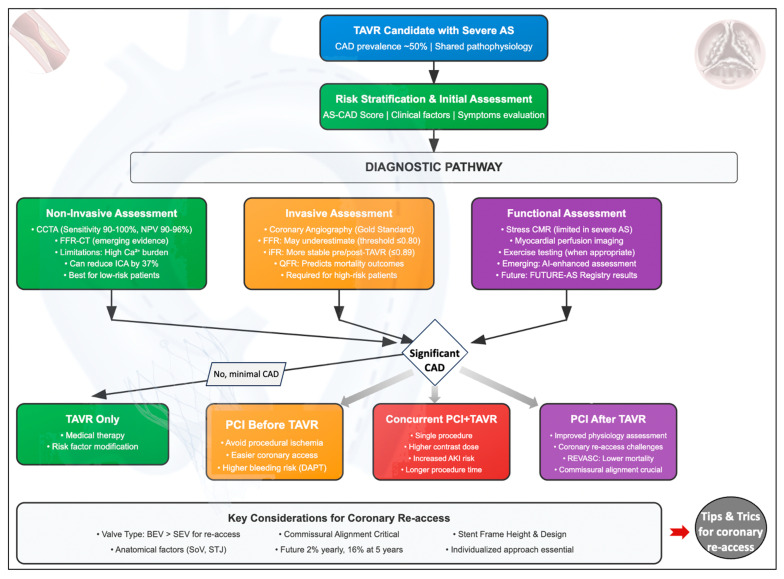
Central figure. AKI: acute kidney injury; AS: aortic stenosis; AS–CAD: aortic stenosis–coronary artery disease; BEV: balloon-expandable valve; CAD: coronary artery disease; CCTA: coronary computed tomography angiography; CMR: cardiac magnetic resonance; DAPT: dual antiplatelet therapy; FFR: fractional flow reserve; FFR-CT: fractional flow reserve-computed tomography; ICA: invasive coronary angiography; iFR: instantaneous wave-free ratio; NPV: negative predictive value; PCI: percutaneous coronary intervention; QFR: quantitative flow ratio; SEV: self-expanding valve; SoV: sinus of Valsalva; STJ: sinotubular junction; TAVR: transcatheter aortic valve replacement.

The aim of this review is to provide a comprehensive overview of the current knowledge regarding the prevalence and clinical impact of CAD in TAVR patients. It will address potential pitfalls in assessing CAD in AS patients using invasive and non-invasive diagnostic tests, while also highlighting emerging techniques that may overcome these limitations. Additionally, the review will discuss the timing of coronary revascularization in relation to the TAVR procedure and approaches for accessing coronary ostia post-TAVR in various scenarios, as well as the assessment and management of CAD in special settings (Figure 1).

## 2. Methods

This narrative review was conducted to synthesize the current evidence on the co-occurrence of AS and CAD, with a focus on challenges surrounding diagnosis and revascularization in patients undergoing TAVR. A comprehensive literature search was performed using the PubMed/MEDLINE, Embase, and Cochrane Library databases. The search strategy included combinations of the following keywords and MeSH terms: aortic stenosis, coronary artery disease, TAVR, PCI, coronary revascularization, coronary angiography, CCTA, FFR, iFR, and valve-in-valve. The time frame for inclusion was 2000–2025. Articles were selected based on relevance, study design, and scientific rigor. Priority was given to randomized controlled trials, large observational registries, and international guideline recommendations. Data were thematically categorized into four main sections: aortic stenosis and coronary artery disease; coronary revascularization before, during, or after TAVR: limits, benefits, and potential solutions; management of coronary artery disease after TAVR; special settings to consider when facing challenges in patients with CAD undergoing TAVR.

## 3. Aortic Stenosis and Coronary Artery Disease

### 3.1. Impact of CAD in Patients with Aortic Stenosis

AS and CAD share common risk factors, and some studies have suggested that they may display similar pathophysiological processes [3], especially in the case of degenerative AS.

Data from a registry evaluating 15,964 patients who underwent TAVR from 2011 to 2013 indicate that the prevalence of CAD is approximately 50%. Among patients with CAD, nearly half show multi-vessel coronary involvement, with around 10% exhibiting left main disease [4]. Additionally, about 20% of the patients undergoing TAVR have a history of prior percutaneous coronary intervention (PCI) [5]. However, it should be acknowledged that the criteria defining CAD varied significantly in the research. On the other hand, few studies have conducted a thorough assessment of CAD severity using angiographic evaluation [1,6], and even fewer have performed hemodynamic assessment using fractional flow reserve (FFR) or instantaneous wave-free ratio (iFR) tools [7,8,9].

The prevalence of CAD reported in different studies can also strongly depend on the patient’s symptoms and the diagnostic methods used for evaluation. Notably, the presence of symptoms has not proven to be a reliable factor in diagnosing CAD in patients with AS, as angina pectoris, dyspnea, and more rarely, syncope, are symptoms common to both conditions. The overlap of these symptoms can potentially mislead the diagnosis, limiting their utility [10]. Hence, coronary angiography is currently considered the gold standard for diagnosing significant coronary lesions before either surgical aortic valve replacement (SAVR) or TAVR.

Nevertheless, computed tomography (CT), which is commonly performed in the preoperative planning for TAVR, has also shown promising results for coronary assessment. In this regard, some authors have suggested performing coronary angiography only in cases where significant lesions are detected on coronary computed tomography angiography (CCTA) or, as is currently common practice, in patients with a low probability of exhibiting CAD. In this setting, a recent study showed excellent performance of CCTA compared to that of angiography, particularly due to its high negative predictive value, despite its low specificity [2]. In addition, CCTA has also proven to be a valuable tool in evaluating segments that have been previously treated with stent implantation [2]. As TAVR expands to include younger patients with AS, CCTA is likely to play an increasingly central role in evaluating CAD. Estimates suggest that the percentage of pre-TAVR coronary angiographies could decrease by 37% due to the growing reliance on CCTA for non-invasive coronary assessment [3]. This shift could accelerate the pre-procedural screening process, allowing it to be conducted mainly as an outpatient procedure and thereby reducing both contrast doses and overall costs.

Nevertheless, the prognostic impact of CAD on patients with AS remains a topic of debate due to conflicting findings in the literature. Patients with CAD undergoing TAVR have been reported to have a 10.1-fold higher risk of 30-day mortality compared to those without CAD [11]. Similarly, data from the Bern TAVR and PCI registry highlighted an increased risk of ischemic events and cardiovascular mortality in TAVR patients with concomitant CAD compared to that for those without CAD at one year follow-up (hazard ratio (HR) 1.86, 95% confident interval (CI) 1.03–3.36; *p* = 0.040) [12]. Notably, a preoperative SYNTAX score >22 has been identified as an independent predictor of all-cause mortality (HR 2.09; *p* = 0.017) [13]. In contrast, other studies suggest a neutral impact of CAD on outcomes following TAVR, including mortality, when adjusted for comorbidities. For instance, the German TAVR registry reported higher crude in-hospital mortality in patients with CAD (10.0% vs. 5.5%, odds ratio (OR) 1.90, 95% CI 1.23–2.93), but this difference became non-significant after adjusting for confounding factors (adjusted OR 1.41, 95% CI 0.85–2.33) [14]. Similarly, the UK TAVR Registry showed no association between CAD presence or extent and early (30-day, *p* = 0.36) or late (4-year, *p* = 0.10) survival after adjustment for confounders in TAVR patients [11]. Moreover, a comprehensive meta-analysis including 2472 patients found that CAD was not a risk factor for increased mortality (OR 1.0, 95% CI 0.67–1.50) [11]. Lastly, in the OBSERVANT II study, a cohort of 1130 patients with CAD was compared to 1505 patients with isolated AS. A propensity score matching process was subsequently performed to create two balanced groups of 813 patients each. At 30 days post-TAVR, no significant differences were observed between the two matched populations with and without CAD regarding mortality (1.7% vs. 2.2%; *p* = 0.480) or major adverse cardiac and cerebrovascular events (MACCE) (3.4% vs. 3.7%; *p* = 0.793). Similarly, at one year, the rates of all-cause mortality, MACCE, and heart failure hospitalizations were comparable between the two groups [15]. However, patients with CAD more frequently underwent repeat PCI due to acute myocardial infarction at one year follow-up, suggesting that, although their overall outcomes in terms of mortality and major complications are similar to those of patients without CAD, they still remain at a greater risk of requiring subsequent coronary interventions [16]. Since studies evaluating the clinical impact of CAD in patients undergoing SAVR or TAVR have produced conflicting results, there is also ongoing debate about whether patients with AS who undergo coronary revascularization gain significant benefits compared to those who receive only valve treatment. For patients who are considered good surgical candidates, SAVR, combined with coronary artery bypass grafting (CABG), represents the standard management strategy when symptomatic AS and CAD coexist. According to the 2021 European Society of Cardiology (ESC)/European Association for Cardiothoracic Surgery Guidelines for the management of valvular heart disease, CABG is recommended for SAVR candidates who display concomitant coronary stenosis of 70% or greater [17]. On the other hand, data on the management of severe CAD among TAVR patients remain limited. Both European and American guidelines recommend performing PCI before TAVR for coronary stenosis of 70% or greater in proximal coronary segments [17,18]. However, this recommendation has a Class IIa indication and a level of evidence C, indicating that it is based on expert consensus rather than high-quality randomized trial data.

In the largest cohort of patients who underwent pre-TAVR PCI published to date, 1197 patients were included, with a total of 1705 coronary lesions treated and a median follow-up of 2 years. Coronary lesions were frequently complex (bifurcation, ostial location, significant calcification), with rotational atherectomy used in 7% of patients. The rate of procedural success (97.3%), intra-stent restenosis (2.3%), and stent thrombosis (0.4%) were comparable to those usually described in “all-comers” PCI. However, the rate of MACCE (death, myocardial infarction, and stroke) was relatively high, with incomplete coronary revascularization (ICR) indicating an increased risk of unfavorable outcomes [3]. Another study involving 1270 patients undergoing TAVR revealed similar findings. Among these patients, 817 patients (64%) showed no CAD, 331 patients (26%) exhibited non-severe CAD, and 122 patients (10%) displayed severe CAD. Over a median follow-up of 1.9 years, 311 patients (24.5%) died. The mortality rate was higher in those with severe CAD and ICR, while in patients with non-severe CAD or “reasonable” ICR, the rate was comparable to that of patients without CAD. After adjusting for multiple variables, the analysis showed that both severe CAD and ICR were independent predictors of increased mortality (HR 2.091, *p* = 0.017; and HR 1.720, *p* = 0.031, respectively). These findings suggest that only severe CAD may significantly affect the outcomes of patients undergoing TAVR [19]. Conversely, evidence from the REVASC-TAVR registry [20] and the ACTIVATION trial [21] provides a more nuanced prospective regarding the role of coronary revascularization in the context of TAVR, which will be discussed further. Lastly, the recent randomized trial NOTION 3 investigated the benefit of coronary revascularization in patients with AS and CAD undergoing TAVR [22]. In this study, 455 patients with severe symptomatic AS and at least one coronary artery stenosis, characterized by a fractional flow reserve of 0.80 or less, or a diameter stenosis of at least 90%, were randomly assigned in a 1:1 ratio to undergo PCI or to receive a conservative treatment, with all patients also undergoing TAVI. At a median follow-up of 2 years, PCI was associated with a lower occurrence of the primary endpoint, defined as a composite of death from any cause, myocardial infarction, or urgent revascularization (26% vs. 36%; HR, 0.71; 95% CI, 0.51 to 0.99; *p* = 0.04). Notably, the benefit of coronary revascularization in TAVR patients was manly driven by a lower incidence of myocardial infarction and urgent revascularization [22].

Furthermore, it is important to highlight that patients with critical left main coronary artery stenosis or high SYNTAX scores were excluded from the previously mentioned randomized trials. To date, this specific patient population has only been included in one propensity score matching analysis, which aimed to compare the outcomes of surgical treatment (SAVR and CABG) with percutaneous treatment (PCI followed by TAVR) in this high-risk cohort. After a median follow-up of 3 years, a comparable rate of MACCE was observed between the two groups. However, the PCI group exhibited a higher risk of requiring repeat PCI procedures [3].

Based on this evidence, the optimal management strategy for patients undergoing TAVR with concomitant CAD remains a subject of debate. Given the conflicting results and the wide variability in the clinical and anatomical presentation of CAD in patients undergoing TAVR, the key to maximizing the benefits of revascularization lies in accurately identifying patients most likely to receive prognostic advantages from PCI. Enhancing a well-defined therapeutic pathway for patients with AS and CAD, especially those treated with TAVR, could significantly streamline their diagnostic and therapeutic journeys. Therefore, a tailored approach that considers individual patient characteristics—such as the extent and severity of CAD, overall comorbidity burden, and procedural risks—could help optimize outcomes. Identifying these patients requires integrating advanced diagnostic tools, risk stratification models, and multidisciplinary decision making, ensuring that revascularization is targeted to those who stand to benefit the most.

In summary, while CAD is prevalent among patients undergoing TAVR, its impact on outcomes remains controversial. Severe CAD and incomplete revascularization are associated with poorer prognosis, but many studies suggest a neutral effect of CAD when adjusted for comorbidities. This underlines the importance of individualized assessment.

#### Pathophysiological Molecular Pathways in the CAD–AS Relationship

AS and CAD indeed share overlapping pathophysiological mechanisms, primarily driven by chronic inflammation, lipid accumulation, endothelial dysfunction, and calcification. These shared pathways not only explain their frequent coexistence but also provide insights into potential therapeutic strategies. At the molecular level, both AS and CAD begin with endothelial injury and dysfunction, both of which increase vascular permeability and promote the expression of adhesion molecules such as VCAM-1 and ICAM-1, facilitating the recruitment of inflammatory cells, including monocytes and T-lymphocytes [23,24]. These cells contribute to foam cell formation through the uptake of oxidized LDL (oxLDL), a hallmark of atherosclerosis that also appears in the early stages of aortic valve disease [24]. Inflammation plays a central role in both conditions, with key pro-inflammatory cytokines such as IL-1β, IL-6, and TNF-α involved in vascular plaque development and valvular interstitial cell (VIC) activation. In AS, these cytokines promote the osteogenic differentiation of VICs, leading to valvular calcification, similar to the process seen in vascular smooth muscle cells within atherosclerotic plaques [25]. Key signaling pathways involved in this process include Notch1, Wnt/β-catenin, and BMP (bone morphogenetic protein) signaling. In particular, loss-of-function mutations in Notch1 have been associated with enhanced calcification in both arterial and valvular tissues [26]. The Wnt/β-catenin pathway, implicated in vascular calcification, also drives VICs toward an osteoblast-like phenotype, further supporting the shared calcific nature of both diseases [27,28]. Additionally, lipoprotein(a) [Lp(a)] has emerged as a common and potent risk factor for both AS and CAD. It contributes to valve and vascular calcification through oxidized phospholipids and inflammatory mediators [29]. This has led to the current interest in targeted therapies such as antisense oligonucleotides against apolipoprotein(a), now being evaluated in clinical trials such as the Lp(a)HORIZON trial [30]. Understanding these shared mechanisms reinforces the need for aggressive risk factor modification and possibly novel anti-inflammatory approaches.

### 3.2. Challenges in Diagnostic Evaluation of Coronary Artery Disease in Patients with AS

#### 3.2.1. Non-Invasive Assessment

In the context of non-invasive assessment of the severity of CAD in patients with AS, various tools can assist in and guide the understanding of its extent. Among these, prediction scores represent a valuable approach, as they help identify patients who are suitable for CCTA instead of coronary angiography prior to TAVR, particularly by focusing on low-risk CAD cases. Dagan et al. developed the AS–CAD score for AS patients to identify those at risk for CAD. The score was created using data from 1782 TAVR patients and includes factors such as gender, prior PCI, stroke, peripheral artery disease (PAD), smoking, diabetes, ejection fraction, and right ventricular pressure. The AS–CAD has demonstrated significant effectiveness in stratifying patients into risk categories, achieving a c-statistic of 0.79 (95% CI 0.74 to 0.84). This tool could potentially reduce the need for coronary angiography by up to 43% in TAVR patients, particularly those at a low risk for CAD [31].

Computed tomography (CT) plays a crucial role in the diagnostic work-up and pre-operative planning of TAVR. Its primary applications include the assessment of vascular access routes, the pathway for the valve delivery system, and the acquisition of key sizing parameters for optimal prosthesis selection [32,33]. Additionally, CCTA has been approved for coronary artery evaluation, leveraging ECG-gated scans to provide detailed information about coronary anatomy without increasing the contrast agent burden. Notably, contrast agents pose a nephrotoxic risk, particularly in elderly patients with impaired kidney function, and it may be even more dangerous when administered via intra-arterial instead of intravenous injection [34,35].

When compared with coronary angiography, CCTA demonstrates high sensitivity and negative predictive value (NPV) for identifying moderate obstructive CAD, defined as stenosis >50% of vessel diameter, in patients undergoing TAVR. The reported sensitivity ranges from 90 to 100%, while the NPV ranges from 90 to 96%. However, its specificity and positive predictive value (PPV) are often suboptimal and vary significantly, with specificity ranging from 37 to 99% and PPV ranging from 37 to 95% [11]. Moreover, a meta-analysis involving 1275 patients undergoing CCTA found a prevalence of CAD that was comparable to that seen in real-world populations undergoing TAVR. The analysis reported a sensitivity of 95%, a specificity of 65%, and an NPV of 94%. Notably, among these patients, 442 patients (35%) were correctly identified as CAD-negative [36]. Similarly, another study confirmed the effectiveness of CCTA in differentiating between patients with no or minimal CAD (indicated by a Coronary Artery Disease-Reporting and Data System (CAD-RADS) score of ≤2) and those with significant CAD (with CAD-RADS score ≥3) [37]. These findings underscore the strength of CCTA as a reliable, non-invasive tool for ruling out significant CAD in TAVR candidates.

The suboptimal specificity of CCTA in patients undergoing TAVR can be attributed to artefacts such as blooming and beam hardening, which result from the high calcium burden commonly found in the coronary arteries of this population cohort [11]. Studies have demonstrated that the higher the coronary calcium score, the greater the risk of false positives and false negatives. In this regard, CCTA exhibited better diagnostic performance in patients with a calcium score < 400 compared to those with a calcium score ≥ 400 [11]. This suggests that coronary calcification is a key factor limiting the accuracy of CCTA, emphasizing the need to interpret the results cautiously when dealing with heavily calcified vessels. For patients with elevated calcium scores, additional imaging or invasive coronary angiography may be necessary to confirm the presence and severity of CAD [11]. Despite these limitations, the increasing use of TAVR in younger patients and those with lower surgical risks suggests that CCTA may play a more central role in evaluating CAD. It has been estimated that the percentage of pre-TAVR coronary angiographies could decrease by 37% due to the growing reliance on CCTA for non-invasive coronary assessments [3]. Furthermore, patients who undergo non-invasive coronary evaluation with CCTA before TAVR experience similar rates of major adverse cardiovascular and cerebrovascular events compared to those assessed with traditional coronary angiography at one year follow-up. These findings indicate that CCTA can be safely and effectively used as the primary test to exclude CAD in the pre-operative management of TAVR, with referrals for invasive coronary angiography only when necessary [38].

Interestingly, advances in computational fluid dynamics, image processing, and artificial intelligence now enable the severity assessment of non-invasive FFR via standard CCTA [39]. Combining anatomical imaging with CCTA and functional assessment using FFR in a single examination could provide a more comprehensive evaluation of CAD, particularly in patients with blooming artifacts (e.g., prior stent implantation) or significant coronary calcifications. This integrated approach could enhance diagnostic accuracy and identify patients who may benefit the most from targeted interventions [40]. However, the evidence supporting physiological assessments in patients with AS is currently limited. Preliminary data are showing promising results, especially in the risk stratification of patients with concomitant CAD [41]. Notwithstanding, it is important to note that the integration of CCTA and FFR in real-world clinical practice remains underexplored, and further research is required to validate its feasibility, reliability, and impact on patient outcomes. In this regard, the FUTURE-AS Registry is an international, multicenter, prospective, open-label study designed to evaluate the efficacy, utility, and accuracy of CCTA and FFR-CT in patients with CAD undergoing TAVR compared to conventional coronary angiography [41]. The registry is currently ongoing, and its results will undoubtedly clarify the utility of CCTA-FFRCT for coronary assessment before TAVR. The pros and cons of the non-invasive techniques are summarized in Table 1A. 

Lastly, among the various imaging modalities used for assessing CAD in patients with AS, another notable technique is cardiac magnetic resonance (CMR). Dobutamine and adenosine stress CMR imaging are well-established and widely utilized methods for diagnosing significant CAD [42]. On the other hand, these procedures are relatively contraindicated in patients with moderate to severe AS. However, a study by Salatski et al. involving 187 patients aimed to evaluate the safety and efficacy of CMR in patients with moderate to severe AS and a high pre-test probability of inducible ischemia, demonstrating that the kinetic abnormalities observed during stress CMR were subsequently explained by coronary angiography, which revealed significant coronary stenoses [43]. Despite these findings, CMR currently does not play a decisive role in determining the diagnostic and therapeutic approach compared to the previously discussed CCTA in AS patients. Further studies with larger sample sizes are essential to enhance its utilization and clinical effectiveness.

In summary, CCTA demonstrates excellent sensitivity and negative predictive value for ruling out obstructive CAD and may reduce the need for invasive angiography in selected patients. However, its diagnostic accuracy is reduced in patients with a high coronary calcium burden.

#### 3.2.2. Invasive Assessment

According to ESC guidelines, invasive coronary angiography (ICA) is recommended before valve surgery in patients with AS who have a history of CAD, suspected myocardial ischemia, left ventricular dysfunction, one or more cardiovascular risk factors, and in postmenopausal women or men older than 40 years old [17]. However, current clinical practice guidelines do not specify the necessity or timing of ICA for patients undergoing TAVR. Some authors propose that pre-TAVR ICA should only be performed when CT suggests significant coronary stenosis. Conversely, other studies concluded that ICA is only necessary for a minority of TAVR candidates, and avoiding it based on CT results does not lead to negative clinical outcomes [38]. Furthermore, in patient with AS, ICA often reveals extensive calcification and tortuosity of the coronary arteries, which can reduce the reliability of angiography in this setting and hinder the accurate assessment of myocardial ischemia among TAVR patients [11].

In this context, the use of hemodynamic monitoring with FFR and iFR has been a topic of controversy regarding its role in guiding decision making for PCI and TAVR [44]. A retrospective analysis by Lunardi et al. involving 216 patients showed that FFR-guided revascularization led to a better MACCE-free survival at 24 months compared to the rates for angiography-guided revascularization (92.6% vs. 82.0%; HR, 0.4; 95% CI, 0.2–1.0; *p* = 0.035). Notably, 78.2% of the intermediate lesions in the FFR group were found to be FFR-negative, according to the conventional cutoff value of 0.80, and these patients were deferred for treatment (10.1161/JAHA.119.012618). In addition, a recent study by Kedhi et al. compared the outcomes of FFR-guided PCI with TAVR against SAVR plus CABG in patients with severe AS and complex CAD. The PCI plus TAVR approach met the non-inferiority criterion and demonstrated superiority in reducing all-cause mortality and life-threatening bleeding compared to the results for SAVR plus CABG [45]. Despite the findings, these pressure-wire indices may be influenced by the hemodynamics associated with AS, which could lead to an underestimation of the true ischemic significance of a coronary obstruction. Moreover, the accuracy of FFR depends on achieving maximal hyperemia, which is typically induced through the intracoronary or intravenous administration of vasodilators [46,47]. Numerous studies have confirmed the safety of using intracoronary and intravenous adenosine [46,47,48]. However, the response to adenosine through the achievement of maximal hyperemia can be affected by several factors in the setting of AS, including left ventricular hypertrophy, elevated ventricular end-diastolic pressure, attenuated flow reserve, negative remodeling of coronary microcirculation, decreased density, and resistance of coronary microvasculature [6,49,50,51]

A recent study by Ahmad et al. demonstrated that both systemic flow and coronary hyperemic flow significantly increased after TAVR when compared with pre-TAVR measurements. However, the flow during the wave-free period of diastole remained unchanged following TAVR. The authors concluded that assessing FFR prior to TAVR may underestimate lesion severity, while iFR appears to be unaffected by the presence of AS [48]. In contrast, an investigation by Yamanaka et al. found a strong correlation between FFR and the presence of ischemia in patients with severe AS and coronary lesions that had been previously assessed using myocardial perfusion scintigraphy. Notably, the optimal FFR threshold was determined to be 0.82, which supports the conclusion that FFR likely underestimates lesion severity in the presence of AS [46]. Moreover, Vendrik et al. reported a significant reduction in FFR values over time, particularly up to 6 months after TAVR, whereas iFR did not show notable variations during the same follow-up period [52]. Similarly, an exploratory study by Scarsini et al. showed that out of 23 coronary lesions, FFR decreased in 3 lesions (13%), while iFR did not show a consistent trend in the long-term after TAVR [53]. Therefore, iFR may be a preferable option for patients with AS, since it does not require adenosine and is independent of systolic flow. However, current evidence is inconclusive, leading to the recommendation to also use conventional thresholds in this setting (FFR ≤ 0.80 and iFR ≤ 0.89) [6]. Recent findings suggest that new parameters are emerging as valuable tools for functional assessment of CAD among patients with AS. In terms of clinical outcomes, the coronary quantitative flow ratio (QFR) has been shown to predict mortality in patients with severe AS treated with TAVR without revascularization. In a retrospective study of 318 patients, 140 patients (44%) presented a diameter stenosis ≥50% in at least one coronary artery, whereas 78 patients (24.5%) had at least one vessel with QFR < 0.80 (positive QFR group) [54]. According to the Kaplan–Meier analysis, patients with positive QFR experienced significantly higher mortality rates during follow-up compared with those without (51.1% vs. 12.1%; *p* < 0.001). In comparison, there was no statistically significant difference in mortality between patients with or without significant CAD as evaluated by angiography (24.3% vs. 19.7%; *p* = 0.244) [54]. The pros and cons of the invasive techniques are summarized in Table 1B.

In summary, hemodynamic indices like FFR and iFR can guide PCI in AS patients but may be influenced by AS-related hemodynamics. iFR appears more stable pre- and post-TAVR, while FFR values may underestimate lesion severity prior to valve intervention. Indeed, while physiological assessment is generally safe for patients with untreated AS, the results may be influenced by the presence of valvular disease. Therefore, caution should be taken in the interpretation of these pressure-wire indices, and the potential benefits of myocardial revascularization should be thoroughly discussed by the heart team. Key studies on the role of functional assessment among patients with AS are summarized in Table 2.

## 4. Coronary Revascularization Before, During, or After TAVR: Limits, Benefits, and Potential Solutions

### 4.1. The Timing Enigma of PCI Strategies in Patients Undergoing TAVR

Although the importance of CAD identification is well known, its treatment in the context of AS remains quite challenging. In the absence of strong, proven evidence, the clinician’s choice is based on factors such as the clinical presentation, patient comorbidities, the complexity of the coronary lesions, and the number of vessels involved. As previously mentioned, current European guidelines [17] for the management of valvular heart disease recommend PCI for patients who primarily require TAVR and display more than 70% stenosis in the proximal segments (Class IIa, level of evidence C). Accordingly, the latest American guidelines recommend performing PCI before TAVR in the context of significant left main or proximal CAD, regardless of the presence of angina [18].

The question is even more challenging in the context of asymptomatic patients. In asymptomatic patients with coexisting AS and CAD, conservative management remains a viable and often necessary strategy, particularly in cases where the severity of stenosis or ischemia does not yet warrant intervention. Current guidelines recommend close surveillance with periodic clinical and echocardiographic follow-up in asymptomatic patients with severe AS and preserved left ventricular function, especially when there is no evidence of high-risk features such as rapid disease progression, elevated biomarkers, or left ventricular dysfunction [17,56]. However, the recently published EARLY TAVR trial showed that among patients with asymptomatic severe AS, a strategy including early TAVR was superior to that of clinical surveillance in reducing the incidence of death, stroke, or unplanned hospitalization for cardiovascular causes [57]. However this situation is more complex in asymptomatic patients with AS and concomitant CAD. Indeed, stable CAD may be managed medically in selected patients without high-risk anatomy or symptoms, as supported by the ISCHEMIA trial, which demonstrated no significant benefit of early invasive intervention over optimal medical therapy in stable CAD [58]. Thus, a carefully individualized, multidisciplinary approach is essential to balance risks and monitor disease progression.

However, following the results of the SURTAVI trial, which showed that complete PCI combined with TAVR can be a viable alternative to SAVR and CABG in patients with severe AS and noncomplex CAD (SYNTAX score ≤ 22), interest in percutaneous revascularization has increased, both in terms of feasibility and efficacy [59]. Various trials assessed the impact of PCI in different scenarios (before, concomitant with, or after TAVR) showing inconsistent results, but suggesting important clinical and prognostic implications [20,59,60,61]). Other studies showed no differences in terms of MACCE between different PCI timing strategies for TAVR candidates [62,63]. However, these findings primarily apply under stable clinical conditions rather than in acute myocardial ischemia settings. Looking ahead, future studies such as the FAITAVI trial and the TAVI PCI trial will evaluate the role of physiology-guided percutaneous revascularization with both FFR and iFR [64,65], while others, such as the COMPLETE TAVR trial, will continue to explore the value of percutaneous revascularization over medical therapy in AS patients with CAD (ClinicalTrials.gov ID NCT04634240). All the main studies, along with their pros and cons in coronary revascularization timing and outcomes among TAVR candidates, are reported in Table 3.

#### 4.1.1. Coronary Revascularization Before TAVR

There are theoretical beliefs suggesting that revascularization before TAVR may offer even greater benefits, but this theory currently lacks strong supporting evidence. Firstly, performing PCI before TAVR can help to avoid ischemia, which may occur during critical phases of the TAVR procedure, such as pacing or valve release. Secondly, accessing the coronary arteries may be easier prior to valve implantation. Several authors have reported a prolonged cannulation time, or even its impossibility, in cases of PCI after TAVR [66]. In addition, although acute coronary syndromes (ACSs) occur in less than 5% of patients following TAVR, the mortality rate associated with myocardial infarction—particularly ST-elevation myocardial infarction—can be notably high, with nearly one-third of patients affected within 30 days. This mortality rate appears to be greater than that observed in alternative settings or in the general population. Additionally, invasive interventions are used less frequently in these cases, with rates below one-third, which may be attributed to the increased difficulty of coronary artery cannulation [67]. Reducing the likelihood of coronary re-entry by treating the disease prior to TAVR may become a therapeutic goal, although new strategies for post-TAVR cannulation are currently being tested to improve operator competence in such situations [68]. However, a sub-analysis of the SURTAVI trial showed no statistically significant differences in clinical outcomes (all-cause mortality or disabling stroke) at 30 days between patients receiving PCI concurrently with TAVR and those receiving PCI up to 7 days before TAVR (7.9% vs. 3.8%; *p* = 0.35) [59]. To reinforce the hypothesis and based on the results of the SURTAVI trial, the ACTIVATION trial, and the NOTION-3 trial were designed [21,22]. One of the main cons in these setting is the bleeding risk. Indeed, factors such as bleeding risk, mainly due to the potential necessity of dual antiplatelet therapy after PCI, should be taken into consideration when deciding to perform PCI in TAVR candidates. In the POPular-TAVI trial, which compared dual antiplatelet therapy (using aspirin and clopidogrel) and aspirin alone, a higher incidence of bleeding was reported with dual antiplatelet therapy [69]. On the other hand, the most common access site in pre-TAVR PCI is the radial site, in contrast to the access site for post-TAVR or concurrent PCI with TAVR. This may partially explain the generally lower rate of major vascular complications observed for the pre-TAVR PCI strategy [20].

Other studies showed conflicting results. The REVASC-TAVI international registry included 1603 patients undergoing TAVI with significant stable CAD, divided into three categories: those who underwent PCI before (65.6%), after (9.8%), or concomitant with (24.6%) TAVR. At 2 years, patients who received PCI following TAVR displayed a considerably lower all-cause death rate than those who underwent PCI before or concurrently with TAVR (6.8% vs. 20.1% vs. 20.6%, respectively; *p* < 0.001) [20]. Moreover, patients who underwent PCI after TAVR exhibited a significantly lower composite endpoint (all-cause death, stroke, MI, or rehospitalization for congestive heart failure) (17.4% vs. 30.4% vs. 30.0% respectively; *p* = 0.003) than that for the other two groups (Table 3). It is of utmost importance to highlight that patients with critical left main coronary artery stenosis or high SYNTAX scores were excluded from the previously mentioned randomized trials. However, these findings suggest that the timing, necessity, and long-term impact of coronary revascularization in the TAVR setting remain areas of active debate. It is plausible that the true clinical effects of PCI prior to TAVI could emerge over a longer follow-up period, emphasizing the need for further studies with extended observation times to clarify the optimal management strategy [16].

#### 4.1.2. Concomitant PCI and TAVR

The feasibility and safety of simultaneous procedures have been demonstrated in several studies [2,16,70,71,72,73]. Using the same arterial access and the resulting reduced costs compared to those for staged procedures would favor concomitant PCI and TAVR. However, the study results are conflicting [20,60,74].

A retrospective analysis of the EVERY-VALVE registry [60] compared the outcomes of concomitant PCI and TAVR with those for a staged approach. The results showed similar rates of technical success, device success, and long-term mortality. However, performing the combined procedure reflects different pros and cons, as shown in Table 3.

On the contrary, the previously mentioned REVASC TAVR registry showed no difference in all-cause mortality between the pre-treatment and concomitant groups [20]. However, accordingly to previous evidence, the volume of contrast used during PCI with TAVR was significantly higher in the concomitant group than in the other two groups (230 mL vs. 110 mL vs. 140 mL, respectively; *p* < 0.001), possibly correlating with the higher rate of acute kidney injury (*p* = 0.011) [20]. Barbanti et al., in a single-center study, analyzed the outcomes of patients undergoing TAVR and PCI in the same session, showing similar composite rate of death, disabling stroke, and MI for patients without CAD and those with severe CAD who did not receive treatment, with no increased procedural risks [75]. However, a meta-analysis of observational studies involving 5000 patients reveals that PCI with TAVI in patients with severe aortic stenosis and concurrent CAD identifies no further therapeutic advantage in terms of relevant patient clinical outcomes when compared to the results for patient undergoing only TAVR [74].

Nevertheless, further studies with long-term follow-up, large-scale cohorts, and randomized trials are necessary to better understand the true impact of concomitant PCI and TAVR on clinical outcomes. Such studies would help clarify the benefits and risks of the combined approach, including its effects on mortality, stroke, and renal function, providing more definitive evidence to guide clinical decision making.

#### 4.1.3. Coronary Revascularization After TAVR

PCI after TAVR can also be a valid option for treating concomitant CAD and AS. Treatment of high-risk ischemic lesions, such as left main or multi-vessel disease, following TAVR does not compromise the success of the procedure. In fact, successful treatment of AS may alleviate left ventricular pressure overload and microvascular dysfunction. This improvement allows for a more accurate physiological assessment of coronary lesion severity and helps identify who would greatly benefit from revascularization [52,76,77]. Ahmad et al. demonstrated how TAVR improves microcirculatory function, regardless of the severity of the underlying CAD [76]. In addition, the earlier resolution of AS may result in better tolerance of procedural complications and pressure drops during PCI, with less impact on organs with low ischemic thresholds such as the kidneys and brain [78].

A recent analysis by Lunardi et al. showed that PCI performed after TAVR does not expose patients to increased risk of peri-procedural hazards and exhibits a trend towards favorable clinical outcomes in the medium to long term [79]. Moreover, the REVASC registry showed that, over a 2-year follow-up period, patients who underwent PCI following TAVR displayed significantly lower rates of all-cause mortality and the composite endpoint (all-cause death, stroke, MI or rehospitalization for heart failure) compared to those who received the treatment either before or concurrently with TAVR [20]. However, the type of valve implanted before or at the time of PCI may limit the generalizability of the results (in most cases, the SAPIEN 3/Ultra balloon-expandable and the Evolut R/PRO self-expandable were used) [20].

Finally, the NOTION-3 trial raises questions concerning the potential mechanisms behind the dislodgement of atherosclerotic plaques following TAVR. Current theories suggest that increased wall stress, potentially linked to patients’ improved exercise capacity after the procedure, could be a contributing factor. Alternatively, it could simply reflect the natural progression of CAD [22]. However, it was observed that patients who underwent PCI experienced a lower risk of a combined outcome, including death from any cause, myocardial infarction, or the need for urgent revascularization, during a median follow-up period of 2 years compared to the results for those who received conservative treatment [22].

Undoubtedly, performing PCI after TAVR presents challenges in re-accessing the coronary arteries, an issue that can be mitigated by ensuring proper commissural alignment (CA) between the new prosthesis and the native aortic valve.

**Table 3:** Timing of PCI in TAVR patients—advantages and disadvantages.

Timing of PCI	PROS	CONS
**BEFORE-TAVR**	**SURTAVI TRIAL** (TAVR and PCI vs. SAVR and CABG) [59] ✓ Similar safety and efficacy outcomes (rates of all-cause mortality or disabling stroke at 2 years). ✓ Lower time needed for the procedure, less time for intensive care, shorter hospitalization.	**SURTAVI TRAIL** (TAVR and PCI vs. SAVR and CABG) ✓ More frequent vascular complications ✓ Acute kidney damage rates higher due to increased contrast load. ✓ Higher rate for new permanent pacemaker implantation.
	**ACTIVATION TRIAL** (PCI prior to TAVR vs. no PCI prior to TAVR) [21] ✓ No evidence for differences in MACCE at 30 days or 1 year (non-inferiority margin was not met).	**ACTIVATION TRIAL** (PCI prior to TAVR vs. no PCI prior to TAVR) ✓ Higher incidence of bleeding at 30 days and 1 year post TAVR-PCI (due to DAPT). ✓ Higher acute kidney damage rate at 30 days and 1 year.
	**NOTION-3** (TAVR + PCI vs. conservative treatment) [22] ✓ Lower risk of composite death from any cause, myocardial infarction, or urgent revascularization at a median follow-up of 2 years.	**NOTION-3** (TAVR + PCI vs. conservative treatment) ✓ Higher bleeding risk.
	**REVASC-TAVI REGISTRY** (PCI before (65.6%), PCI after (9.8%), or PCI concomitant (24.6%) with TAVR) [20] ✓ Lower rate of major vascular complications (radial site access). ✓ Low rate of myocardial infarction.	**REVASC-TAVI REGISTRY** (PCI before (65.6%), PCI after (9.8%), or PCI concomitant (24.6%) with TAVR) ✓ Higher incidence of disabling stroke, all-cause death, and composite endpoint (stroke, MI, or HF rehospitalization) at 2 years.
**CONCOMITANT-TAVR**	**SURTAVI TRIAL** (TAVR and PCI vs. SAVR and CABG) [59] ✓ Similar safety and efficacy outcomes (rates of all-cause mortality or disabling stroke at 2 years). ✓ Lower time needed for the procedure, less time for intensive care, shorter hospitalization. ✓ Clinical outcomes were similar at 30 days, but concomitant PCI resulted in fewer complications and more procedural efficiency (sub analysis: staged PCI vs. concomitant PCI).	**REVASC-TAVI REGISTRY** (PCI before (65.6%), PCI after (9.8%), or PCI concomitant with (24.6%) TAVR) [20] ✓ Higher rate of acute kidney injury. ✓ Higher incidence of disabling stroke, all-cause death and composite endpoint (all cause death, stroke, MI or HF rehospitalization) at 2 years ✓ Higher rate of in-hospital mortality.
	**REVASC-TAVI REGISTRY** (PCI before (65.6%), PCI after (9.8%), or PCI concomitant (24.6%) with TAVR) [20] ✓ Low rates of HF rehospitalization.	
	**EVERY-VALVE REGISTRY** (77.6% no PCI, 10.1% stepwise PCI, 12.3% concomitant PCI) [60] ✓ Reduction in the number of puncture wounds for vascular access. ✓ Increased patient compliance (single hospitalization). ✓ Economic advantages regarding materials and equipment. ✓ Similar length of stay in intensive care unit.	**EVERY-VALVE REGISTRY** (77.6% no PCI, 10.1% stepwise PCI, 12.3% concomitant PCI) ✓ Longer procedure time. ✓ Higher risk of bleeding (associated with anticoagulant and antithrombotic therapy). ✓ Higher risk of acute kidney injury (higher contrast dose).
	**BARBANTI ET AL.** [66] ✓ Similar composite rate of death, disabling stroke, and MI. ✓ No increased procedural risks.	
**AFTER-TAVR**	**REVASC-TAVI REGISTRY** (PCI before (65.6%), PCI after (9.8%), or PCI concomitant (24.6%) with TAVR) [20] ✓ Lower rates of all-cause mortality and composite endpoint (all-cause death, stroke, MI or rehospitalization for heart failure) at 2 years. ✓ Adequate physiological assessment of coronary lesion severity.	**REVASC-TAVI REGISTRY** (PCI before (65.6%), PCI after (9.8%), or PCI concomitant with (24.6%) to TAVR) ✓ Higher rate of MI and HF rehospitalization.
	**LUNARDI ET AL.** [79] ✓ No exposure to increased risk of peri-procedural hazards. ✓ Favorable outcomes in the medium to long term.	

CABG: coronary artery by-pass grafting; DAPT: dual antiplatelet therapy; HF: heart failure; MACCE: major adverse cerebro-cardiovascular events; MI: myocardial infarction; PCI: percutaneous coronary intervention; SAVR: surgical aortic valve replacement; TAVR: transcatheter aortic valve replacement; NOTION-3: Nordic Aortic Valve Intervention Trial 3.

### 4.2. The Importance of Commissural Alignment During TAVR Procedure

Coronary obstruction or difficult coronary access after transcatheter prostheses implantation are well-documented complications. During TAVR or valve-in-valve procedures (TAVR in surgical valve replacement or TAVR in TAVR), these issues might be firstly caused by the displacement of the native or bioprosthetic aortic valve leaflets by the new transcatheter heart valve (THV), which may be pinned in an open position, thus potentially obstructing coronary ostia or making coronary access more challenging. However, it may also result from commissural misalignment, which poses a risk of overlap between the neo-commissures and the native coronary ostia. Therefore, CA, referring to the angular relationship between the native commissures and the bioprosthetic commissures following the implantation of a surgical or transcatheter biological prosthesis, is essential for reducing the risk of coronary occlusions and ensuring future coronary access, as well as for redo TAVR procedures, especially in patients with longer life expectancy [80]. Moreover, a proper CA has also demonstrated a favorable impact on valve function and durability [81]. On the other hand, a potentially unfavorable alignment may be associated with abnormal fluid flow patterns across the new prosthesis, increasing leaflet stress and potentially leading to blood stagnation inside the sinus of Valsalva and consequent thrombosis [81].

**Figure 2:**
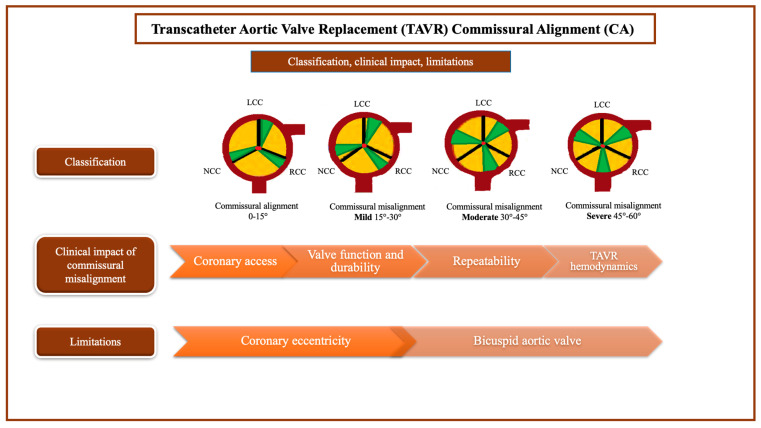
Commissural alignment and related factors. CA: commissure alignment; LCC: left coronary cusp; NCC: non coronary cusp; RCC: right coronary cusp; TAVR, transcatheter aortic valve replacement.

Recently, CA was defined by the ALIGN-TAVR (commissures aortic valve alignment) consortium using a four-level scale, as follows: aligned (0°–15°), slightly misaligned (15°–30°), moderately misaligned (30°–45°), severely misaligned (45°–60°) [82]. While CT scans are the most reliable method for evaluating the commissural alignment between native valve commissures and valve-specific markers, the correct post-procedural alignment is often verified using fluoroscopy during TAVR procedures. This is because using CT scans can be more time-consuming and costly. [80]. Commissural alignment definition and its main impacting factors are presented in Figure 2.

Regardless of definition, coronary misalignment (CMA) remains a frequent occurrence in TAVR patients. Fuchs et al. found that only 22% of THVs were correctly aligned in a series of 212 patients [83]. Notably, 31% of the prostheses exhibited severe CMA. Furthermore, the study revealed that the degree of commissural alignment did not correlate with transvalvular gradients or simulated coronary filling. However, moderate or severe CMA was significantly associated with a higher incidence of mild central aortic regurgitation compared to the results for patients with mild or less misalignment (7.8% vs. 1.1%; *p* = 0.03) [83].

To the best of our knowledge, there are currently no official guidelines that provide instructions for using TAVR devices to achieve neo-commissural alignment. Furthermore, the aortic anatomy and the orientation of each patient’s native aortic valve vary. Therefore, it is essential to develop a specific method tailored to each individual to ensure accurate commissural alignment during THV implantation [84]. Current techniques for THVs alignment should rely on fluoroscopy, CT imaging, and THV-specific technologies that provide valve markers to guide peculiar maneuvers during implantation to achieve CA [85].

## 5. Management of Coronary Artery Disease After TAVR

**Figure 3:**
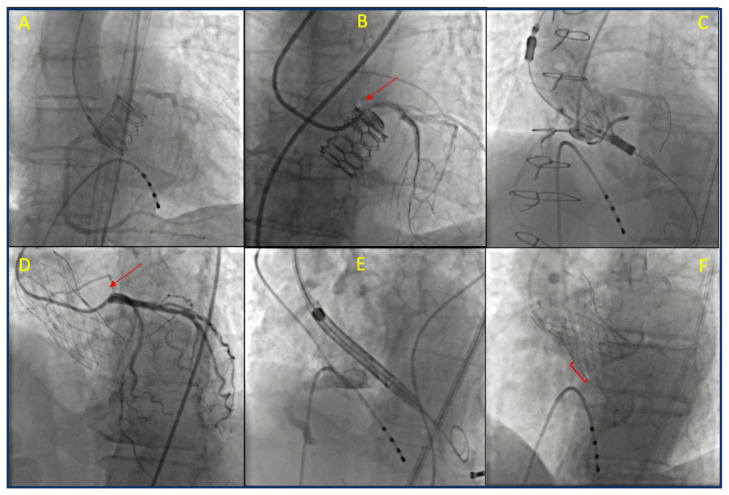
Angiographic visualization of the spatial relationships between percutaneous prostheses and coronary arteries in the periprocedural phase or after deployment. (**A**) Aortography following implantation of a balloon expandable Myval Octacor prosthesis, demonstrating patency of both coronary arteries. (**B**) Selective cannulation of the left coronary artery (red arrow) in a patient with a Myval Octacor valve. (**C**) Valve-in-valve implantation of an ACURATE neo2 device (the large open-cell design of the lower stent frame reduces the risk of coronary obstruction). (**D**) Cannulation of the left coronary artery (red arrow) in a patient implanted with a Portico valve. (**E**) Angiographic guidance during positioning of a self-expanding valve, emphasizing the balance between implantation depth and coronary height. (**F**) Final angiogram after Evolut valve implantation; the red-shaded area indicates implantation depth—greater depth reduces the risk of coronary obstruction but may increase the likelihood of conduction disturbances.

With the expansion of TAVR to include low-risk and younger patients, an increasing number of individuals face the potential risk of requiring future percutaneous procedures to treat coronary or valvular diseases. The lifetime risk of needing unplanned PCI after TAVR is low for patients without CAD at the time of the procedure. However, this risk increases over time for those with pre-existing CAD, particularly in patients with multivessel disease and a high SYNTAX score [86]. Data from the literature and registries indicate that coronary angiography and PCI may be necessary for nearly 2% of patients undergoing TAVR within the first year and for 16% within five years post-TAVR. Moreover, approximately two-thirds of these procedures are performed in the context of acute coronary syndromes, mainly unstable angina or NSTEMI [87,88]. Additionally, younger patients are considered a high-risk group due to their longer life expectancy, as are those with multiple CAD risk factors and those who have a history of PCI [89,90]. Given this context, it is essential to carefully plan the initial valve procedure, while also considering anatomical and technical factors that may affect the success and prognosis of any future procedures. Ensuring the possibility of coronary re-access after TAVR is a critical consideration in this planning process. Angiographic visualization of transcatheter aortic valve replacement periprocedural images and their spatial relationship with the coronary arteries are reported in Figure 3.

### 5.1. Factors Influencing Coronary Re-Access After TAVR

**Figure 4:**
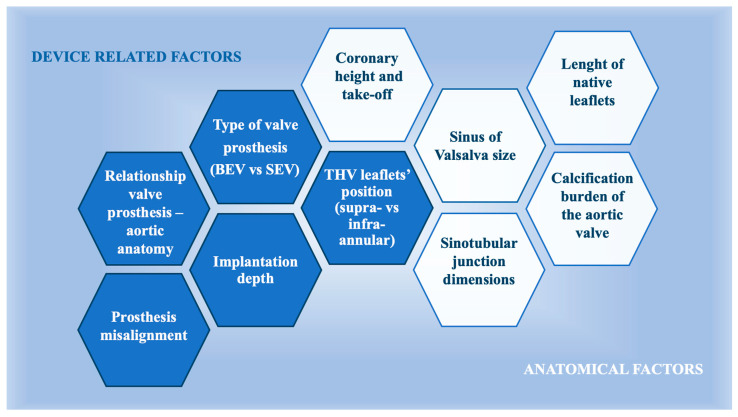
Main factors that impact the feasibility of coronary re-access after TAVI: the relationship between device related factors and anatomical factors. BEV, balloon-expanding valve; SEV, self-expanding valve; THV, transcatheter heart valve.

The feasibility of accessing coronary arteries after TAVR is influenced by several key factors (Figure 4). These include the spatial relationship between the prosthetic valve and the aortic wall, the height of the stent frame, the positioning of the bioprosthetic leaflets, the final location of the bioprosthetic commissures in relation to the coronary ostia, and the specific design and type of the prosthetic valve [91]. On the other hand, coronary access after TAVR is also influenced by several other critical anatomical factors, such as the size of the sinuses in relation to the prosthetic valve size, the height and width of the sinotubular junction, and the location of the coronary arteries, particularly their height and relationship to the native commissures [6]. There is increasing evidence suggesting that high stent-frame THVs with a supra-annular leaflet position present more challenges for selective coronary cannulation, especially in cases of severe commissural misalignment or high implant positioning [92].

The design of the TAVR prosthesis used during implantation has an important impact on the feasibility of subsequent coronary access. Generally, TAVR devices with shorter prosthesis frames, such as balloon-expandable valves, facilitate easier coronary re-access. In contrast, self-expanding supra-annular valves, characterized by longer prosthesis frames, a supra-annular leaflet position, and smaller frame cells, pose greater challenges to coronary access [93,94,95]. This is particularly true when attempting to cannulate the right coronary artery (RCA). Kim et al. examined the feasibility of coronary angiography or PCI among 449 patients in the acute setting following TAVR. The success rate for coronary angiography of the RCA was higher in patients with a short stent-frame prosthesis (99.6% vs. 95.9%; *p* = 0.005). However, the success rates for left coronary artery (LCA) cannulation were similar across both groups (99.7% vs. 98.7%; *p* = 0.24). Thus, the success rate for PCI was 91.4%, with no significant difference between patients with short or long stent-frame prostheses (90.4% vs. 93.4%; *p* = 0.44) [96]. Conversely, the REVIVAL trial reported a high success rate (96.6%) for unplanned PCI, with no significant differences between patients that underwent balloon-expandable or self-expandable TAVR device implantation (100% vs. 94.9%; *p* = 0.150) [97]. However, focusing on the peculiarities of different valve prostheses is important in the assessment of patients with CAD and AS.

### 5.2. Coronary Re-Access in Self-Expanding Valves (SEVs)

Earlier smaller studies indicated that the CoreValve prosthesis (Medtronic) could present challenges for coronary cannulation after TAVR [98]. The Evolut devices feature a supra-annular design, which extends above the coronary ostia, and an external pericardial skirt added to the Evolut PRO and PRO+ devices that reduces paravalvular leakage (PVL) [99]. The RE-ACESS trial, a prospective registry that included 300 patients who underwent coronary access before and after TAVR with various THVs (123 Evolut R/Pro, 96 Sapien 3/Ultra, 72 ACURATE, and 9 Portico) reported a successful coronary cannulation in 92.3% of cases. However, unsuccessful cannulation occurred mostly with Evolut R/Pro valves (22/23 failed attempts). Key independent factors predicting failure of coronary cannulation included a large TAVR-to-sinus of Valsalva (SoV) ratio, the use of Evolut R/Pro valves, and a shallow implantation depth of the TAVR devices [66]. Therefore, ensuring the proper sizing and alignment of the prosthesis is crucial to prevent these issues. It is essential that the SEV is appropriately sized relative to the patient’s annular dimensions to avoid excessive deployment depth and to maintain coronary access [6]. Conversely, factors such as valve oversizing relative to SoV dimensions and a higher implantation depth (<6 mm), which is typical of certain TAVR approaches aimed at minimizing permanent pacemaker implantation (PPI), may increase the likelihood of failed coronary cannulation [66]. For this reason, in situations where there is concern about coronary obstruction—especially when the coronary ostia measure less than 12 mm and the diameter of the SoV is less than 30 mm [100]—using a slightly deeper depth of approximately 5 mm may enhance access to the coronary arteries. However, this approach may increase the risk of PPI and PVL. As a result, commissural alignment, as previously reported, has become a significant aspect of TAVR. If a commissural post happens to be positioned directly in front of a coronary ostium, the catheter must then enter through the valve from a cell superior and/or lateral, making co-axial and co-aligned coronary engagement extremely difficult [101,102].

To achieve a proper alignment with the Evolut valve, the delivery catheter should be inserted with the flushing flash port positioned at 3 o’clock relative to the descending aorta. The valve also features a hat marker located 90 degrees counterclockwise from the C paddle, which aligns with the right coronary cusp (RCC) and the left coronary cusp (LCC) commissure of the prosthesis. Placing this hat marker on the outer curvature of the descending aorta during valve navigation, prior to reaching the final position, is recommended. This maneuver is designed to position the hat marker toward the outer curvature or the anterior center in the three-cusp coplanar view, which is focused on the right coronary cusp. Additionally, the marker is oriented to the anterior center in the view where the right and left coronary cusps overlap. This technique successfully implemented CA in 85.9% of patients (n = 64) in the ALIGN-ACCESS study [82]. To further address the issue of coronary re-access, the new Evolut FX plus features a revolutionary design enhancement, with larger diamond-shaped windows for coronary access, spaced at 120 degrees apart, and incorporates three radiopaque markers to further enhance positioning accuracy and commissural alignment. This improvement facilitates access without compromising radial strength or valve performance. By following the implantation steps to achieve commissural alignment, the alignment of the diamond-shaped windows with the coronary arteries can also be enhanced, making future coronary re-access easier [103].

On the other hand, the ACURATE neo (Boston Scientific) features a distinct nitinol structure compared to that of the Evolut devices, although both exhibit a supra-annular design. The device’s stabilization arches promote axial self-alignment within the native annulus, and its large open cells facilitate easy access to the coronary arteries. However, higher implantation depth may still pose challenges for future coronary re-access, and commissural alignment could help mitigate these issues. According to the ALIGN TAVI trial, when the commissural post was misaligned, the rate of coronary artery overlap was 28.0% with the left main artery (LM), 42.0% with RCA, and 51.0% with one or both coronary arteries.

Conversely, proper commissural alignment, when the commissural post was at the center back and the inner curve at initial deployment, resulted in significantly lower overlap rates [82].

The ACURATE neo2’s relatively easy torquing ability helps achieve consistent commissural alignment, which is important for coronary access. In a three-cusp view, the optimal orientation is reached with a 1:1:1 orientation of stent posts and two visible free cells. In the cusp overlap (R-L) view, the best orientation displays a single post and a free cell on the inner curve (2:1 alignment). If misalignment occurs, torquing can be applied, and this technique appears effective and safe to achieve commissural alignment [85]. In the ALIGN-ACCESS study, aligning the supra-annular SEV led to a higher success rate for selective coronary angiography, with lower contrast use and shorter fluoroscopic time compared to the results for misaligned devices [104]. Moreover, the newer ACURATE Prime device offers an improved design with predictable commissural alignment, additional markers, and quick deployment, potentially improving coronary re-access and redo procedures, although further randomized trials are necessary to confirm these benefits. The Portico valve (Abbott) is a fully recapturable, repositionable, and retrievable nitinol SEV with a porcine pericardial sealing cuff and intra-annular bovine pericardial leaflets. Its larger cells (compared to those of Evolute) and intra-annular leaflet positioning enhance coronary access after TAVR, with the lower commissural position facilitating coronary re-engagement, unless a commissural post directly obstructs a coronary ostium. Nevertheless, a recent study indicated that severe commissural misalignment and coronary overlap affected more than half of patients receiving the Portico THV. In many of these cases, at least one coronary ostium was obstructed by a closed cell, complicating coronary access post-TAVR [105]. Proper alignment during implantation (one of these three radiopaque commissural posts must be isolated and aligned at the right of the screen in the cusp overlap view in order to achieve THV implantation with neo-commissural alignment) is crucial for minimizing these complications. However, the COMALIGN trial found that the posts were often poorly visible in Portico THVs, leading to a higher incidence of commissural misalignment compared to the results for other devices [84]. As a response, the Navitor Vision, a newer version of the Portico system, features an active sealing cuff to reduce PVL and three well-visualized radiopaque markers for accurate depth measurement (at 3 mm), improving coronary re-access and valve performance. Its wide cell geometry, intra-annular leaflet design, and lowest neoskirt height (23 mm) provide for unhindered coronary access. Further research will provide information regarding the implications of the Navitor valve in terms of coronary re-access and redo procedures. Future studies, including the Comfort study (ClinicalTrials.gov ID: NCT05779787), will further explore the effects of random vs. aligned Navitor valve implantation for coronary re-access. Other SEVs available in certain countries, such as Hydra and Allegra, also offer promising features. Hydra’s design includes tentacle-like components for better alignment and large open cells (more than 15 Fr) for coronary access. At the same time, Allegra uses large, diamond-shaped cells and radiopaque markers to facilitate valve positioning and coronary re-engagement. However, data on coronary re-access with these devices is still limited due to the lack of large, randomized trials [106].

### 5.3. Coronary Re-Access in Balloon-Expandable Valves

Due to their smaller radial profile, BEVs are less likely to obstruct coronary ostia. Notable examples of BEVs include the SAPIEN XT and SAPIEN 3 (Edwards Lifesciences), both of which feature an intra-annular and sub-coronary design. Their low-profile stent frame, composed of 12 open cells in the upper section, facilitates coronary access. Coronary cannulation can typically be achieved either above the outflow of the valve or through the large cells in the upper row of the stent. This depends on the THV’s implantation depth relative to the patient’s anatomical features. However, challenges can arise, particularly in cases where voluminous and long leaflets extend beyond the inner skirt, especially in patients with small sinuses. In such instances, the commissural tabs might obstruct the coronary ostia, making it difficult to achieve catheter co-axial and co-aligned engagement [94].

Several studies have confirmed that BEVs are associated with fewer complications related to coronary access compared to SEVs. For instance, in the RE-ACCESS trial, 96 patients (32%) were treated with SAPIEN 3 and SAPIEN Ultra valves, showing that these valves rarely interfere with coronary arteries [107]. Ochiai et al. also identified a significantly higher success rate of selective cannulation following TAVR with intra-annular BEVs than with SEVs (BEVs, 85.9%, vs. SEVs, 43.8%) [108]. These findings suggest that BEVs provide a more favorable environment for future coronary re-access. However, BEVs are not completely free of risks for coronary obstruction. Rogers et al. reported that coronary access post-TAVR with SAPIEN 3 can be challenging in 9% to 13% of patients, and where the commissural post will land in relation to the coronary ostia is unpredictable [109]. The latest SAPIEN 3 valve features a more streamlined profile and an outer polyethylene terephthalate sealing cuff, which is designed to improve paravalvular sealing compared with that noted for earlier versions of the SAPIEN valve [110]. However, this valve is taller, which may occasionally cause it to extend above the coronary ostia, potentially obstructing coronary access. On the other hand, the cells in the upper row of the SAPIEN 3 valve are 38% larger in area compared to those in the SAPIEN XT valve [94], although, among the 12 open cells of this prosthesis, three contain the commissures and leaflet attachments. This arrangement poses the possibility that a commissure could end up directly in front of a coronary ostium after valve deployment. Another advantage of the SAPIEN 3 prosthesis is that the implantation height can be precisely controlled using the central radiopaque marker, which corresponds to the center of the delivery balloon. This feature aids in the accurate positioning of the crimped valve. In this regard, the diameter and height of the STJ are critical for SAPIEN 3 implantation. In patients with a smaller STJ diameter and lower height, there is an increased risk of complications when attempting future coronary access above the valve [94]. However, it is important to note that predicting commissural alignment before deployment of Sapien BEVs is not possible. This unpredictability makes achieving consistent commissural alignment challenging. However, after THVs positioning, the neo-commissures can be individualized by three “double lines” between the hexagonal crowns in the upper cells. Additionally, crimping the SAPIEN 3 valve in specific orientations does not appear to affect commissural alignment [82]. The SAPIEN 3 Ultra RESILIA valve is a newer version of the SAPIEN 3, designed to improve valve longevity and reduce the need for reintervention through proprietary calcium-blocking technology. The S3 Ultra valve displays similar procedural complications to those of the original SAPIEN 3 but exhibits lower rates of PVL and improved valve performance, as demonstrated in the OCEAN-TAVR registry [111]. Given its potential for long-lasting durability, trials are necessary to individuate its impact on future coronary re-access.

In contrast, the Myval valve (Meril) is constructed with a hybrid honeycomb structure made from cobalt alloy, characterized by open cells (more than a half at the aortic end and less than a half at the ventricular end), enhancing the annular radial force and guaranteeing easier coronary re-access [112]. The Myval valve is available in a range of nine sizes, allowing for selection of the most suitable size for different native annulus shapes. An alternating dark and light band-like pattern created during crimping acts as a reference point to locate and deploy the THV over the native annulus. As advanced version, the Myval Octacor THV, features a design with two rows of octagonal cells [113]. As a sub-coronary BEV, it offers higher success rates for coronary re-access compared to those for SEVs. However, misalignment can occasionally impede coronary flow and access after TAVR. To address this, the OctaAlign technique aligns the commissures of the Myval Octacor with the patient’s native aortic valve by positioning one commissure in the mirror image of the mid-RCC, according to CT analysis, which will deploy the valve anatomically towards the RCC non-coronary cusp (NCC) commissure. This approach helps minimize misalignment, reduces the risk of coronary artery obstruction, optimizes future coronary access after valve implantation, and reduces complications during repeat procedures [114]. However, further studies are needed to assess its role in coronary re-access and redo TAVR procedures.

### 5.4. Tips and Tricks for Coronary Ostia Cannulation

**Figure 5:**
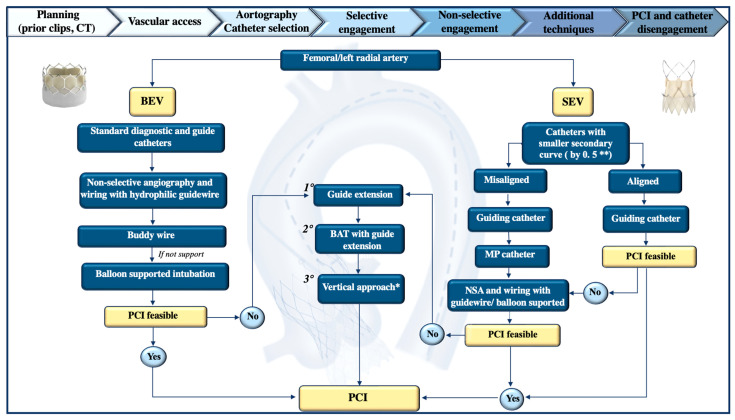
Coronary ostia cannulation algorithm in patient with prior transcatheter aortic valve implantation. Main procedural steps of coronary ostia cannulation in the non-acute setting include planning, choosing vascular access, eventual aortography and consequent catheter selection, tentation for selective engagement, otherwise non-selective engagement, and additional techniques as reported in the algorithm below. Following successful PCI, the catheter should be carefully and gently disengaged, ideally over the wire, to minimize kinking or valve damage. Based on the type of prothesis (balloon-expanding or self-expanding) and good alignment or misalignment, additional catheters or techniques may be required. * Vertical approach with Judkins Left 6, narrow mammary, and snake sinus has been described as useful for the Accurate Valve. In rare cases, they may be applied in balloon expanding valves. ** Judkins Left 3.0 or 3.5, Judkins Right 4.0, Left Femoral 3.0/3.5, or Ikari right 1.0/1.5 for left coronary ostium. Otherwise use guiding catheter Judkins Left or extra-back-up (EBU) 3.0/3.5. For right coronary ostium cannulation, use JR 3.5/4, femoral right or Amplatz right (sometimes oversizing by 0.5 may be helpful in cases of wide sinus of Valsalva). BAT = balloon-assisted tracking; BEV = balloon-expanding valve; CT = computed tomography; MP = multipurpose; NSA = non-selective angiography; PCI = percutaneous coronary intervention; SEV = self-expanding valve.

Post-TAVR coronary cannulation can be difficult due to subsequent structural modifications caused by the presence of the THV prosthesis that may lead to coronary obstruction or displacement of coronary ostia. Achieving successful coronary access is particularly challenging with older generations of valves, in centers without TAVR experience, or for operators with limited expertise. We propose a detailed algorithm we believe to be helpful for coronary cannulation following TAVR (Figure 5). Cannulation is generally easier with BEVs due to their sub-coronary positioning compared to that of SEVs [107]. As previously discussed, the difficulty of engaging the coronary ostia depends on various anatomical and technical factors. These include the anatomy of the aortic root and STJ, the dimensions of the sinuses, the height and location of coronary artery take-offs, the design and positioning of THVs, and the alignment of the valve’s commissures [6]. This latter issue is crucial, since misalignment can interfere with accessing the ostia, increasing the risk of procedural complications [6]. In this context, a comprehensive pre-procedural imaging strategy, including contrast-enhanced CT scans, can assist in planning coronary access in non-acute situations.

A CT scan evaluates the aortic root, valve characteristics, and the relationship between the coronary ostia and the THV, identifying any potential obstacles such as valve frames, thrombosis, or calcifications [115]. It can also provide details on vascular access options. If a CT scan is unavailable, due to factors like chronic kidney disease or the risk of procedure delays, if the TAVR procedure was performed at the same center, reviewing clips from previous procedures can offer valuable insights regarding anatomical alignment, valve depth, and coronary take-off positions. In the absence of prior clips, an aortogram should be performed, typically using a left oblique view or a right caudal view for certain SEVs like the Evolute valve. Moreover, choosing the most familiar vascular access for the operator is crucial, keeping in mind both valve characteristics and the anatomical configuration of the vessels. For instance, access through tortuous brachiocephalic vessels should be avoided. The right radial artery remains the preferred access for most operators in everyday cath lab procedures, although its use in post-TAVR patients has not been extensively studied. Most previous studies on coronary re-access in TAVR patients have been conducted using femoral access [104,107,116]. An alternative to femoral access is left radial artery access, which may reduce catheter manipulation and improve angulation for selective coronary cannulation. Moreover, studies suggest that selective engagement of the coronary ostia might be more successful by employing additional techniques, such as using a coronary guidewire or guide-extension catheter, particularly when using radial access [104,107].

As previously noted, the type of valve can significantly influence catheter selection and procedural steps. For patients with BEVs, standard diagnostic catheters could be used without modification just by positioning them over the superior edge of the valve frame. In some instances, a hydrophilic guidewire can be carefully advanced under fluoroscopy to avoid damaging the valve, or a buddy wire may be used for additional support. In contrast, SEVs, especially when misaligned, complicate coronary cannulation. Procedural failures may occur due to difficulties in crossing the valve struts or incorrect placement within the coronary artery resulting from inadequate catheter support [87]. Yudi et al. proposed a catheter selection strategy based on the type of THV, the specific procedure being performed (coronary angiography or PCI), and the relationship between the transcatheter commissural post and the coronary ostium. In challenging cannulation scenarios involving BEVs, especially when a commissural tab obstructs the coronary ostium—non-selective angiography may be sufficient. Alternatively, a guidewire “fishing” technique, followed by the positioning of the guide catheter, or the use of catheter extensions can also be effective. In rare cases of high BEV deployment, a multipurpose catheter may facilitate engagement [94].

For SEVs, the constrained design of the valve frame can reduce the size of the ascending aortic root compared to the native aortic root. This reduction often requires the use of a catheter with a smaller secondary curve to engage the left coronary ostium. For example, a Judkins Left (JL) 3.5 catheter may be preferred over a JL4 catheter. Indeed, it is generally recommended to reduce catheter size by 0.5 for LCA cannulation compared to the standard catheter size typically used for this access. In cases of well-aligned valves, the operator should choose a catheter shape that facilitates a perpendicular approach to the valve frame and coaxial alignment with the ostium. While valves like Accurate and Portico generally feature larger cells, Evolute valves may pose more challenges. Typically, the JL catheter is the first choice for LCA cannulation, with secondary options including Judkins Right (JR) 4, Ikari Right 1.5–1.0, or femoral left 3.0–4.0 catheters [94]. For RCA cannulation, the JR4 catheter is preferred. However, longer-tipped catheters like AR2, JR4.5, or Ikari Right guides may be better suited for engaging wide SoV. In cases of misalignment, a multipurpose catheter may be helpful. Moreover, engaging the cell near the commissural post, usually through non-selective cannulation with a hydrophilic wire, can assist in gaining coronary access, occasionally with the support of a balloon. Once the coronary artery is engaged, the operator should attempt selective cannulation by advancing a 0.035-inch wire into the THV near the nadir of the cusps, using a slow push-and-pull technique to align with the THV cell strut closest to the coronary ostium. However, cannulation with SEVs is often non-selective, so employing a guiding catheter can enhance stability, especially when the 0.035-inch wire is retained inside during contrast injection. If selective cannulation is still not feasible, advancing the wire distally into the coronary vessel can assist in positioning the catheter. Even when non-selective, the catheter remains stable enough to perform PCI, as the metallic THV frames provide sufficient support. If additional support is needed, balloon anchoring can improve catheter selectivity. If cannulation remains difficult due to misalignment or high THV positioning, guide extensions or balloon-assisted tracking (BAT technique) can help improve coaxial alignment and coronary ostium engagement [117,118]. In cases of severe commissural misalignment, particularly with the Acurate Neo valve, three novel techniques—vertical approach with JL 6, narrow mammary, and snake sinus—have been proposed to externally bypass obstructive elements of the valve frame [119]. However, with the advent of newer valve designs, such as the Evolute Fx Plus and Accurate Prime, along with improvements in operator skills and commissural alignment, coronary re-access is likely to become easier. However, larger trials are needed to confirm these trends.

Given the challenges of coronary re-access in patients with severe AS and established or intermediate CAD, particularly in younger, lower-risk patients, a multidisciplinary heart team approach is essential for managing CAD in these cases.

## 6. Special Settings to Consider When Facing Challenges in Patients with CAD Undergoing TAVR

### 6.1. Insight into Coronary Access in TAVR-in-SAVR and TAVR-in-TAVR

There is growing concern regarding the potential for widespread failure of bioprosthetic valves, particularly as younger patients increasingly receive these implants. The stented bioprosthetic valve is the most frequently used type for surgical implantation. In the context of TAVR-in-SAVR, the leaflets of the surgical aortic valve are displaced, creating a cylinder-like effect that leads to sinus sequestration and coronary obstruction. This obstruction is more commonly observed in patients undergoing valve-in-SAVR procedures compared to those undergoing initial TAVR, with registry data (such as from the VIVID registry) showing obstruction rates between 2.4% and 3.5% [120]. Factors that increase the risk of impaired coronary access include female sex, small coronary ostia (<10 mm), reduced sinus size (<30 mm), short distances between the valve and coronary arteries (<4 mm), and smaller STJ (<2 mm) [120,121]. In addition, both stentless bioprostheses and stented bioprostheses with externally mounted valve leaflets have been identified as independent risk factors for coronary obstruction. Consequently, it is essential to accurately measure the annular plane, coronary artery heights, STJ height, and SoV dimensions. These measurements should be followed by the implantation of a “virtual valve” to estimate the distances between the valve and coronary arteries (VTC) and between the valve and STJ (VTSTJ) [120,122]. Notably, each prosthetic valve model has a manufacturer-specified size; however, this does not reflect the actual internal diameter (ID) of the prosthesis. The ID of the stent frame is measured separately from that of the prosthesis and varies depending on the type of stented SAV and the method used to attach the tissue leaflets to the stent frame [123]. To assess the risk of coronary obstruction, a CT scan should be performed, and the VTC and VTSTJ distances should be measured. Distances smaller than 3 mm are considered to indicate a high risk of coronary obstruction [123]. Therefore, it is important to select an appropriate second valve, ensure proper commissural alignment, and consider utilizing new techniques, such as balloon valve fracture and remodeling, to prevent coronary obstruction and coronary re-access problems. Although procedural guidelines for engaging the coronary arteries in this setting are not well-established, the previously mentioned techniques may provide significant benefits. 

Extending TAVR indications to lower-risk and younger patient populations will also likely result in an increase in redo-TAVR procedures in the future. Treating structural degeneration of THVs by implanting a second THV is technically feasible, although there is insufficient strong data supporting this approach. Concerns still remain regarding valve durability but also concerning the risk of coronary artery obstruction and the ability to access the coronary ostia if PCI is needed in the future [124,125]. In these patients, coronary re-access is more complex than in those undergoing a single TAVR procedure due to the additional risk posed by the displaced leaflets of the native aortic valve, as well as the presence of two metallic frames and a greater likelihood of commissural suture posts obstructing access to the coronary ostia. In such cases, a small STJ and supra-annular devices are linked to an increased risk of impaired coronary access [126]. Therefore, it is crucial to understand the three-dimensional interaction between the degenerated THV, the coronary ostia, and the aortic root, which can guide pre-procedural planning for TAVR-in-TAVR procedures. After a TAVR-in-TAVR procedure, coronary access may be unfeasible in more than 30% of patients currently undergoing TAVR. As previously mentioned, particularly those with a small STJ and those who receive supra-annular THVs are at high risk for impaired coronary access after redo-TAVR [127]. In these cases, novel techniques such as the BASILICA technique (bioprosthetic or native aortic scallop intentional laceration to prevent iatrogenic coronary artery obstruction) may be less effective when the neo-commissures of the THV are not aligned with those of the native aortic valve. Consequently, Tarantini et al. introduced the concept of the risk plane (RP), which defines the level below which the passage of a coronary catheter becomes impossible after implantation of the second valve [128]. CT analysis is essential for assessing the valve-to-aorta distance (VTA), which refers to the gap between the prosthesis frame at the RP level and the aortic wall. A free space of more than 2 mm is required to allow navigation of a 6 French catheter behind the prosthesis struts and to engage the coronary ostium. According to their findings, coronary access following TAVR-in-TAVR is primarily feasible in two scenarios: the first scenario occurs when the coronary ostia and access are situated above the RP (type 1 scenario), and the second one (type 2a), when the coronary ostia are located below the RP, but the access remains above the RP, with a VTA greater than 2 mm [128].

Accordingly, the choice of the first THV can significantly influence the likelihood of coronary access impairment after TAVR-in-TAVR. This is because different prostheses have varying neoskirt heights [6]. Meier et al. demonstrated that different redo TAVR combinations can result in a wide range of neoskirt heights, which do not all offer the same prospects for future coronary re-access. Specifically, using a tall frame valve in a failed tall frame valve with misaligned cells may severely complicate coronary cannulation. Conversely, in their study, an Accurate neo implanted in a Sapien valve resulted in the largest and most favorable combination of cells for catheter access [95]). However, if coronary obstruction is expected after redo-TAVR, chimney stenting can be employed to restore coronary blood flow in cases of impending or established coronary artery occlusion. Even so, future access to the coronary arteries after chimney stenting can be quite challenging [129]. Recently, evidence has shown that a modified version of heterotopic chimney stenting during valve-in-valve TAVR can lead to successful end-on cannulation through the stent ostium [130]. Despite these advancements, our understanding of this area remains limited.

### 6.2. Future Directions

CA is required in TAVR for successful coronary access and for potential future redo-TAVR procedures. Moreover, CA may greatly impact valve function and duration. However, in some cases, coronary eccentricities are present. Recent studies indicate that coronary eccentricity—where the coronary ostium is not centered in the corresponding cusp—is more commonly observed with the RCA. This can result in coronary overlap, despite adequate commissural alignment [131]. Consequently, the previously discussed techniques for CA may not be sufficient in these cases. To address this issue, new approaches have been proposed using CT-derived views to better understand the relationship between TVH and coronary ostia ratios. Hence, promising studies suggest focusing on coronary alignment rather than on just commissural alignment [132]. Notably, a recent CT study has shown that cusp asymmetry occurs more frequently in patients with type 1 bicuspid aortic valves (69%) compared to those with tricuspid aortic valves (17.5%). This indicates that achieving CA may be more difficult with bicuspid aortic valves, making coronary alignment a potentially more accurate approach [132].

Regarding post-TAVR antithrombotic therapy, while multiple studies have provided valuable insights, several questions remain unanswered. First, the optimal duration of oral antithrombotic therapy is not yet well established. Second, further studies are needed to test the use of oral anticoagulant therapy (OAC) and investigate whether lower doses could reduce adverse hemorrhagic effects while maintaining the same effectiveness, especially in young people undergoing TAVR. Currently, the ACASA-TAVI randomized trial (Anticoagulation vs. Acetylsalicylic acid after TAVI) (NCT05035277) is underway. This study compares OAC with DOAC (rivaroxaban, 20 mg per day; apixaban, 5 mg BID; or edoxaban, 60 mg per day) against single antiplatelet therapy, according to the work in ref. [133]. Another ongoing study is the AVATAR (Anticoagulation Only Vs. Anticoagulation and Aspirin after TAVI) (NCT02735902) study, which is recruiting TAVR patients with a baseline indication for long-term OAC. This study aims to assess the net clinical benefit of OAC (vitamin K antagonist or DOAC) compared to that of OAC with aspirin at 12 months.

#### The Role of Sodium-Glucose Cotransporter-2 Inhibitors in TAVR Patients

Patients undergoing TAVR exhibit significant comorbidities, leading to the high risk of periprocedural complications. This increases the risk of re-hospitalization for these patients. The grounds for readmission are split between non-cardiac and cardiac causes. Among the causes of heart disease, heart failure is the main reason for re-hospitalization [134]. Post-TAVR medical therapy could reduce the incidence of rehospitalizations. In this regard, it is known that sodium-glucose cotransporter-2 inhibitors (SGLT2is) significantly improve cardiovascular and renal outcomes in patients with type 2 diabetes mellitus (T2DM) and in non-diabetic patients with heart failure [135,136]. Furthermore, recent studies have shown that in patients with severe AS, the SGLT2 protein is expressed in human cardiomyocytes, and in particular, this protein is overexpressed in the subgroup of patients with reduced left ventricular ejection fraction (LVEF < 50%), regardless of glucose metabolic control [137]. Based on these observations, Paolisso et al. hypothesized that SGLT2is could promote favorable cardiac remodeling in patients with T2DM suffering from severe AS, LVEF < 50%, and extra-valvular cardiac damage (EVCD) undergoing TAVR [138]. Based on glucose-lowering therapy at hospital discharge, patients were stratified in SGLT2is vs. no-SGLT2is users. The primary endpoint was a composite of all-cause death and HF hospitalization (MACE) at 2-year follow-up. Secondary outcomes included all-cause death, cardiovascular death, and HF hospitalization. At the end of the study, in diabetic patients with severe AS, LVEF < 50%, and EVCD undergoing TAVI, the use of SGLT2is was associated with a more favorable cardiac remodeling and a reduced risk of MACE at 2-year follow-up [138]. Another factor that increases re-hospitalizations post TAVR is acute kidney injury (AKI). AKI in patients undergoing TAVR has been shown to significantly worsen results, increasing mortality in both the short and long term [139]. Although SGLT2is are known to improve renal and cardiovascular outcomes in patients with T2DM [140,141], as well as to produce nephroprotective effects in chronic kidney disease (CKD), even in patients without T2DM, little information is available regarding their nephroprotective effects in patients undergoing TAVR. This information would be particularly relevant in patients with concomitant CAD, where the use of contrast agents for both TAVR and coronary revascularization increases the risk of contrast-induced nephropathy [142]. In this setting, a recent study showed that, compared to standard glucose-lowering therapies, novel antidiabetic agents, including SGLTis, significantly reduce the incidence of contrast-associated acute kidney injury among diabetic patients undergoing PCI [143]. Moreover, a recent trial conducted by Paolisso et al. analyzed the risk of AKI after TAVR in patients with T2DM with or without CKD, comparing those treated with SGLT2is (users of SGLT2is) vs. those receiving other anti-diabetic agents (no-SGLT2is users). They have shown that in diabetic patients with CKD undergoing TAVR, SGLT2is therapy was associated with a lower occurrence of AKI compared to that for those not treated with SGLT2is, suggesting a potential nephroprotective effect in this high-risk population [144]. The use of SGLT2is in treatment protocols for patients undergoing TAVR who present critical clinical characteristics could help reduce the risk of heart failure and AKI, particularly in patients with concomitant CAD. Although current data are promising, they should be considered as hypotheses, as large-scale randomized controlled trials are needed to definitively establish the role of SGLT2is in the prevention of adverse events after TAVR. Such studies could clarify the optimal timing, dosage, and patient selection criteria.

## 7. Conclusions

In recent years, the management of patients with both AS and CAD has advanced significantly, largely due to the rapid adoption of TAVR in younger and low-risk patients. The coexistence of both conditions has important prognostic and clinical implications, as untreated CAD can negatively impact clinical outcomes. Diagnosing CAD in the context of TAVR is particularly challenging and requires a personalized approach that combines both non-invasive and invasive methods to assess the severity and extent of coronary disease. While hybrid procedures and coronary revascularization during TAVR show promise, determining the optimal timing for PCI remains an area of ongoing investigation. Additionally, re-accessing the coronary arteries after TAVR presents technical challenges, particularly when using different valve types (SEVs or BEVs), which can affect future interventions. Advanced imaging, careful valve sizing, commissural alignment, and specific techniques are essential to ensure future coronary access. In special cases, such as TAVR-in-TAVR or TAVR-in-SAVR procedures, these challenges are compounded by the need for meticulous planning and risk assessment, particularly concerning the initially implanted valve. An individualized antithrombotic therapy adds another layer of complexity, as considerations such as atrial fibrillation, advanced age, and bleeding risks must be taken into account. However, new device designs, emerging technologies such as QFR and CT-FFR, as well as future studies focusing on coronary alignment may offer promising tools for evaluating and managing CAD in TAVR patients. Although many questions remain unanswered, ongoing research and technological advancements are expected to refine clinical practices in the future.

## Figures and Tables

**Table 1:** Comparison of non-invasive and invasive techniques in diagnostic evaluation of coronary artery disease in patients with aortic stenosis undergoing TAVR.

A. Non-Invasive Assessment	PROS	CONS
**Prediction scores**	Stratifying patients into risk categories.	More useful for low risk of CAD.
**CCTA**	Safety; detailed information about coronary artery; high sensitivity and NPV for moderate obstructive CAD.	Nephrotoxic risk; often suboptimal specificity and PPV for high calcium Burden.
**Non-invasive FFR from standard CCTA**	Integrated approach; useful in patients with blooming artifacts and significant calcification.	Limited evidence; underexplored in real world clinical practice.
**CMR**	Good evaluation of kinetic abnormalities and EF%.	Limited evidence; relative contraindications in AS patients.
**B. Invasive Assessment**	**PROS**	**CONS**
**ICA**	Gold standard to guide decision making and time for revascularization.	Invasive procedure; nephrotoxic risk; radiation; low reliability in extensive calcification and tortuosity of the coronary arteries.
**FFR and iFR**	Functional assessment; improves decision making for revascularization.	Invasive procedure; radiation; underestimation of the true ischemic significance because of AS hemodynamics; time and costs.

AS: aortic stenosis; CAD: coronary artery disease; CCTA: coronary computed tomography angiography; CMR: cardiac magnetic resonance; EF: ejection fraction; FFR: fractional flow reserve; iFR: instantaneous wave-free ratio; ICA: invasive coronary angiography; NPV: negative predictive value; PPV: positive predictive value; TAVR: transcatheter aortic valve replacement.

**Table 2:** Invasive physiological assessment of coronary lesions using FFR or iFR in patients undergoing TAVR.

Study	Study Design	Cohort	Inclusion Criteria	Results and Conclusions
Yamanaka et al. [46]	Observational study aim: to compare iFR values vs. FFR values vs. adenosine SPECT.	95 patients	Severe AS + intermediate coronary artery stenosis (116 vessels).	Good correlation between iFR and FFR (R = 0.854; *p* < 0.0001); an optimal cutoff of 0.82 for the iFR to indicate an FFR ≤ 0.75.
Scarsini et al. [53]	Observational study aim: to investigate variations of FFR and iFR at baseline and after TAVI.	14 patients	Severe AS + physiology assessment of coronary lesions at baseline immediately after TAVR and at follow-up (14–29 months).	FFR decreased in 3 patients (13%), while iFR did not show a systematic trend at long-term after TAVI.
Ahmad et al. [48]	Prospective study aim: to evaluate variations in FFR and iFR after TAVI.	28 patients	Severe AS + physiology assessment of coronary artery disease (30 lesions) at rest and during hyperemia immediately before and after TAVR.	Systemic flow and coronary hyperemic flow increased significantly after TAVR: reduction in FFR value mean, while iFR did not change after TAVR.
Vendrik et al. [52]	Prospective study aim: to investigate variations in FFR and iFR after TAVI (6 months).	13 patients	Lower-risk patients with TAVI and moderate to severe coronary lesions.	Significant reduction in FFR values, whereas iFR did not show significant variations.
Lunardi et al. [55]	Retrospective study aim: to compare angiography-guided vs. FFR-guided revascularization in patients undergoing TAVI.	216 patients	Severe AS undergoing TAVI + bystander CAD (30–70%).	FFR-guided revascularization presented a better MACE-free survival at 24 months, and bystander intermediate coronary lesions were FFR-negative in 78.2% of cases.

AS: aortic stenosis; CAD: coronary artery disease; iFR: instantaneous wave-free ratio; FFR: fractional flow reserve; TAVR: transcatheter aortic valve replacement; MACE: major adverse cardiovascular event; TAVI: Transcatheter Aortic Valve Implantation; SPECT: Single Photon Emission Computed Tomography.

## Data Availability

Not applicable.
